# Identifying significant edges in graphical models of molecular networks

**DOI:** 10.1016/j.artmed.2012.12.006

**Published:** 2013-03

**Authors:** Marco Scutari, Radhakrishnan Nagarajan

**Affiliations:** a Genetics Institute, University College London, Darwin Building, Gower Street, WC1E 6BT London, United Kingdom; b Division of Biomedical Informatics, Department of Biostatistics, College of Public Health, University of Kentucky, 725 Rose Street, Multidisciplinary Science Bldg, 230F, Lexington, KY 40536-0082, USA

**Keywords:** Graphical models, Bayesian networks, Model averaging, *L*_1_ norm, Molecular networks

## Abstract

**Objective:**

Modelling the associations from high-throughput experimental molecular data has provided unprecedented insights into biological pathways and signalling mechanisms. Graphical models and networks have especially proven to be useful abstractions in this regard. Ad hoc thresholds are often used in conjunction with structure learning algorithms to determine significant associations. The present study overcomes this limitation by proposing a statistically motivated approach for identifying significant associations in a network.

**Methods and materials:**

A new method that identifies significant associations in graphical models by estimating the threshold minimising the *L*_1_ norm between the cumulative distribution function (CDF) of the observed edge confidences and those of its asymptotic counterpart is proposed. The effectiveness of the proposed method is demonstrated on popular synthetic data sets as well as publicly available experimental molecular data corresponding to gene and protein expression profiles.

**Results:**

The improved performance of the proposed approach is demonstrated across the synthetic data sets using sensitivity, specificity and accuracy as performance metrics. The results are also demonstrated across varying sample sizes and three different structure learning algorithms with widely varying assumptions. In all cases, the proposed approach has specificity and accuracy close to 1, while sensitivity increases linearly in the logarithm of the sample size. The estimated threshold systematically outperforms common ad hoc ones in terms of sensitivity while maintaining comparable levels of specificity and accuracy. Networks from experimental data sets are reconstructed accurately with respect to the results from the original papers.

**Conclusion:**

Current studies use structure learning algorithms in conjunction with ad hoc thresholds for identifying significant associations in graphical abstractions of biological pathways and signalling mechanisms. Such an ad hoc choice can have pronounced effect on attributing biological significance to the associations in the resulting network and possible downstream analysis. The statistically motivated approach presented in this study has been shown to outperform ad hoc thresholds and is expected to alleviate spurious conclusions of significant associations in such graphical abstractions.

## 1 Introduction and background

Graphical models [1,2] are a class of statistical models which combine the rigour of a probabilistic approach with the intuitive representation of relationships given by graphs. They are composed by a set **X** = {*X*_1_, *X*_2_, …, *X*_*N*_} of *random variables* describing the quantities of interest and a *graph* G=(V,E) in which each *node* or *vertex* v∈V is associated with one of the random variables in **X** (they are usually referred to interchangeably). The *edges e* ∈ *E* are used to express the dependence relationships among the variables in **X**. The set of these relationships is often referred to as the *dependence structure* of the graph. Different classes of graphs express these relationships with different semantics, which have in common the principle that graphical separation of two vertices implies the conditional independence of the corresponding random variables [2]. The two examples most commonly found in literature are *Markov networks* [3,4], which use undirected graphs, and *Bayesian networks* (BNs) [5,6], which use directed acyclic graphs.

In principle, there are many possible choices for the joint distribution of **X**, depending on the nature of the data. However, literature has focused mostly on two cases: the *discrete case* [3,7], in which both **X** and the *X*_*i*_ are multinomial random variables, and the *continuous case* [3,8], in which **X** is multivariate normal and the *X*_*i*_ are univariate normal random variables. In the former, the parameters of interest are the *conditional probabilities* associated with each variable, usually represented as conditional probability tables; in the latter, the parameters of interest are the *partial correlation coefficients* between each variable and its neighbours (i.e. the adjacent nodes in G).

The estimation of the structure of the graph G is called *structure learning* [1,4], and involves determining the graph structure that encodes the conditional independencies present in the data. Ideally it should coincide with the dependence structure of **X**, or it should at least identify a distribution as close as possible to the correct one in the probability space. Several algorithms have been presented in the literature for this problem, thanks to the application of many results from probability, information and optimisation theory. Despite differences in theoretical backgrounds and terminology, they can all be grouped into only three classes: *constraint-based algorithms*, that are based on conditional independence tests; *score-based algorithms*, that are based on goodness-of-fit scores; and *hybrid algorithms*, that combine the previous two approaches. For some examples see Bromberg et al. [9], Castelo and Roverato [10], Friedman et al. [11], Larrañaga et al. [12] and Tsamardinos et al. [13].

On the other hand, the development of techniques for assessing the statistical robustness of network structures learned from data (e.g. the presence of artefacts arising from noisy data) has been limited. Structure learning algorithms are commonly studied measuring differences from the true (known) structure of a small number of reference data sets [14,15]. The usefulness of such an approach in investigating networks learned from real-world data sets is limited, since the true structure of their probability distribution is unknown.

A more systematic approach to model assessment, and in particular to the problem of identifying statistically significant features in a network, has been developed by Friedman et al. [16] using bootstrap resampling [17] and model averaging [18]. It can be summarised as follows:

1. For *b* = 1, 2, …, *m*:
  (a) sample a new data set ${\text{X}}_{b}^{*}$ from the original data **X** using either parametric or nonparametric bootstrap;
  (b) learn the structure of the graphical model $G_{b}=(\text{V},E_{b})$ from ${\text{X}}_{b}^{*}$.
2. Estimate the probability that each possible edge *e*_*i*_, *i* = 1, …, *k* is present in the true network structure $G_{0}=(\text{V},E_{0})$ as
  (1) $$\hat{P}(e_{i})=\frac{1}{m}\sum_{b=1}^{m}1_{\left\{e_{i}\in E_{b}\right\}},$$
  where $1_{\left\{e_{i}\in E_{b}\right\}}$ is the indicator function of the event {*e*_*i*_ ∈ *E*_*b*_} (i.e. it is equal to 1 if *e*_*i*_ ∈ *E*_*b*_ and 0 otherwise).

The empirical probabilities $\hat{P}(e_{i})$ are known as *edge intensities* or *arc strengths*, and can be interpreted as the degree of *confidence* that *e*_*i*_ is present in the network structure $G_{0}$ describing the true dependence structure of **X** .^1^ However, they are difficult to evaluate, because the probability distribution of the networks $G_{b}$ in the space of the network structures is unknown. As a result, the value of the confidence threshold (i.e. the minimum degree of confidence for an edge to be significant and therefore accepted as an edge of $G_{0}$) is an unknown function of both the data and the structure learning algorithm. This is a serious limitation in the identification of significant edges and has led to the use of ad hoc, pre-defined thresholds in spite of the impact on model assessment evidenced by several studies [16,19]. An exception is Nagarajan et al. [20], whose approach will be discussed below.

Apart from this limitation, Friedman's approach is very general and can be used in a wide range of settings. First of all, it can be applied to any kind of graphical model with only minor adjustments (for example, accounting for the direction of the edges in BNs, see Section 4). No distributional assumption on the data is required in addition to the ones needed by the structure learning algorithm. No assumption is made on the latter, either, so any score-based, constraint-based or hybrid algorithm can be used. Furthermore, parallel computing can easily be used to offset the additional computational complexity introduced by model averaging, because bootstrap is embarrassingly parallel.

In this paper, we propose a statistically motivated estimator for the confidence threshold minimising the *L*_1_ norm between the cumulative distribution function (CDF) of the observed confidence levels and the CDF of the confidence levels of the unknown network $G_{0}$. Subsequently, we demonstrate the effectiveness of the proposed approach by re-investigating two experimental data sets from Nagarajan et al. [20] and Sachs et al. [21].

## 2 Selecting significant edges

Consider the empirical probabilities $\hat{P}(e_{i})$ defined in Eq. (1), and denote them with $\hat{\text{p}}=\{{\hat{p}}_{i},\;i=1,\ldots ,k\}$. For a graph with *N* nodes, *k* = *N*(*N* − 1)/2. Furthermore, consider the order statistic

(2) $${\hat{\text{p}}}_{\left(\cdot \right)}=({\hat{p}}_{\left(1\right)},{\hat{p}}_{\left(2\right)},\ldots ,{\hat{p}}_{\left(k\right)})\;\mathrm{with}\;{\hat{p}}_{\left(1\right)}⩽{\hat{p}}_{\left(2\right)}⩽\cdots ⩽{\hat{p}}_{\left(k\right)}$$

derived from $\hat{\text{p}}$. It is intuitively clear that the first elements of ${\hat{\text{p}}}_{\left(\cdot \right)}$ are more likely to be associated with non-significant edges, and that the last elements of ${\hat{\text{p}}}_{\left(\cdot \right)}$ are more likely to be associated with significant edges. The ideal configuration ${\tilde{\text{p}}}_{\left(\cdot \right)}$ of ${\hat{\text{p}}}_{\left(\cdot \right)}$ would be

(3) $${\tilde{p}}_{\left(i\right)}=\left\{\begin{array}{cc} 1 & \mathrm{if}\;e_{\left(i\right)}\in E_{0}\\ 0 & \mathrm{otherwise} \end{array}\right),$$

that is the set of probabilities that characterises any edge as either significant or non-significant without any uncertainty. In other words,

(4) $${\tilde{\text{p}}}_{\left(\cdot \right)}=\{0,\ldots ,0,1,\ldots ,1\}.$$

Such a configuration arises from the limit case in which all the networks $G_{b}$ have exactly the same structure. This may happen in practice with a consistent structure learning algorithm when the sample size is large [22,23].

A useful characterisation of ${\hat{\text{p}}}_{\left(\cdot \right)}$ and ${\tilde{\text{p}}}_{\left(\cdot \right)}$ can be obtained through the empirical CDFs of the respective elements,

(5) $$F_{{\hat{\text{p}}}_{\left(\cdot \right)}}(x)=\frac{1}{k}\sum_{i=1}^{k}1_{\left\{{\hat{p}}_{\left(i\right)}<x\right\}}$$

and

(6) $$F_{{\tilde{\text{p}}}_{\left(\cdot \right)}}(x)=\left\{\begin{array}{cc} 0 & \mathrm{if}\;x\in (-\infty ,0)\\ t & \mathrm{if}\;x\in [0,1)\\ 1 & \mathrm{if}\;x\in [1,+\infty ) \end{array}\right).$$

In particular, *t* corresponds to the fraction of elements of ${\tilde{\text{p}}}_{\left(\cdot \right)}$ equal to zero and is a measure of the fraction of non-significant edges. At the same time, *t* provides a threshold for separating the elements of ${\tilde{\text{p}}}_{\left(\cdot \right)}$, namely

(7) $$e_{\left(i\right)}\in E_{0}\Leftrightarrow {\tilde{p}}_{\left(i\right)}>F_{{\tilde{\text{p}}}_{\left(\cdot \right)}}^{-1}(t)$$

where $F_{{\tilde{\text{p}}}_{\left(\cdot \right)}}^{-1}(t)=\underset{x\in \mathbb{R}}{\inf}\{F_{{\tilde{\text{p}}}_{\left(\cdot \right)}}(x)⩾t\}$ is the *quantile function* [24].

More importantly, estimating *t* from data provides a statistically motivated threshold for separating significant edges from non-significant ones. In practice, this amounts to approximating the ideal, asymptotic empirical CDF $F_{{\tilde{\text{p}}}_{\left(\cdot \right)}}$ with its finite sample estimate $F_{{\hat{\text{p}}}_{\left(\cdot \right)}}$. Such an approximation can be computed in many different ways, depending on the norm used to measure the distance between $F_{{\hat{\text{p}}}_{\left(\cdot \right)}}$ and $F_{{\tilde{\text{p}}}_{\left(\cdot \right)}}$ as a function of *t*. Common choices are the *L*_p_ family of norms [25], which includes the Euclidean norm, and Csiszar's *f*-divergences [26], which include Kullback–Leibler divergence.

**Fig. 1:**
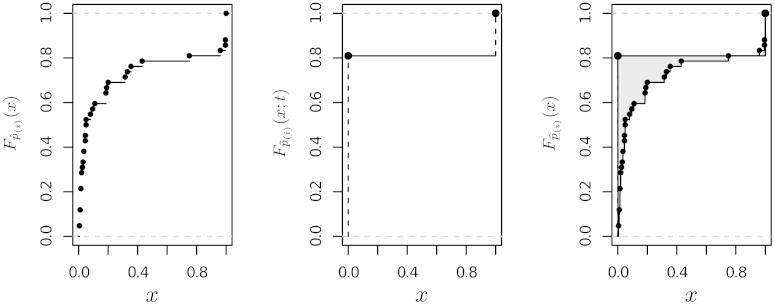
The empirical CDF $F_{{\hat{\text{p}}}_{\left(\cdot \right)}}$ (left), the CDF $F_{{\tilde{\text{p}}}_{\left(\cdot \right)}}$ (centre) and the *L*_1_ norm between the two (right), shaded in grey.

The *L*_1_ norm

(8) $$L_{1}(t;{\hat{\text{p}}}_{\left(\cdot \right)})=\int |F_{{\hat{\text{p}}}_{\left(\cdot \right)}}(x)-F_{{\tilde{\text{p}}}_{\left(\cdot \right)}}(x;t)|\mathrm{dx}$$

appears to be particularly suited to this problem; an example is shown in Fig. 1. First of all, note that $F_{{\hat{\text{p}}}_{\left(\cdot \right)}}$ is piecewise constant, changing value only at the points ${\hat{p}}_{\left(i\right)}$; this descends from the definition of empirical CDF. Therefore, for the problem at hand Eq. (8) simplifies to

(9) $$L_{1}(t;{\hat{\text{p}}}_{\left(\cdot \right)})=\sum_{x_{i}\in \{\{0\}\cup {\hat{\text{p}}}_{\left(\cdot \right)}\cup \{1\}\}}|F_{{\hat{\text{p}}}_{\left(\cdot \right)}}(x_{i})-t|(x_{i+1}-x_{i}),$$

which can be computed in linear time from ${\hat{\text{p}}}_{\left(\cdot \right)}$. Its minimisation is also straightforward using linear programming [27]. Furthermore, compared to the more common *L*_2_ norm

(10) $$L_{2}(t;{\hat{p}}_{\left(\cdot \right)})=\int {\left[F_{{\hat{p}}_{\left(\cdot \right)}}(x)-F_{{\hat{p}}_{\left(\cdot \right)}}(x;t)\right]}^{2}dx$$

or the *L*_∞_ norm

(11) $$L_{\infty }(t;{\hat{\text{p}}}_{\left(\cdot \right)})=\max_{x\in [0,1]}\{|F_{{\hat{\text{p}}}_{\left(\cdot \right)}}(x)-F_{{\tilde{\text{p}}}_{\left(\cdot \right)}}(x;t)|\},$$

the *L*_1_ norm does not place as much weight on large deviations compared to small ones, making it robust against a wide variety of configurations of ${\hat{\text{p}}}_{\left(\cdot \right)}$.

Then the identification of significant edges can be thought of either as a *least absolute deviations estimation* or an *L*_1_ *approximation* of the form

(12) $$\hat{t}=\underset{t\in [0,1]}{\mathrm{argmin}}\;L_{1}(t;{\hat{\text{p}}}_{\left(\cdot \right)})$$

followed by the application of the following rule:

(13) $$e_{\left(i\right)}\in E_{0}\Leftrightarrow {\hat{p}}_{\left(i\right)}>F_{{\hat{\text{p}}}_{\left(\cdot \right)}}^{-1}(\hat{t}).$$

Note that, even though edges are individually identified as significant or non-significant, they are not identified independently of each other because $\hat{t}$ is a function of the whole ${\hat{\text{p}}}_{\left(\cdot \right)}$.

A simple example is illustrated below.

Example 1 Consider a graphical model based on an undirected graph G with node set **V** = {*A*, *B*, *C*, *D*}. The set of possible edges of G contains 6 elements: (*A*, *B*), (*A*, *C*), (*A*, *D*), (*B*, *C*), (*B*, *D*) and (*C*, *D*). Suppose that we have estimated the following confidence values:

(14) $${\hat{p}}_{\mathrm{AB}}=0.2242,\;{\hat{p}}_{\mathrm{AC}}=0.0460,\;{\hat{p}}_{\mathrm{AD}}=0.8935,\;{\hat{p}}_{\mathrm{BC}}=0.3921,\;{\hat{p}}_{\mathrm{BD}}=0.7689,\;{\hat{p}}_{\mathrm{CD}}=0.9439.$$

Then ${\hat{\text{p}}}_{\left(\cdot \right)}=\{0.0460,0.2242,0.3921,0.7689,0.8935,0.9439\}$ and

(15) $$F_{{\hat{\text{p}}}_{\left(\cdot \right)}}(x)=\left\{\begin{array}{cc} 0 & \mathrm{if}\;x\in (-\infty ,0.0460)\\ \frac{1}{6} & \mathrm{if}\;x\in [0.0460,0.2242)\\ \frac{2}{6} & \mathrm{if}\;x\in [0.2242,0.3921)\\ \frac{3}{6} & \mathrm{if}\;x\in [0.3921,0.7689)\\ \frac{4}{6} & \mathrm{if}\;x\in [0.7689,0.8935)\\ \frac{5}{6} & \mathrm{if}\;x\in [0.8935,0.9439)\\ 1 & \mathrm{if}\;x\in [0.9439,+\infty ) \end{array}\right).$$

**Fig. 2:**
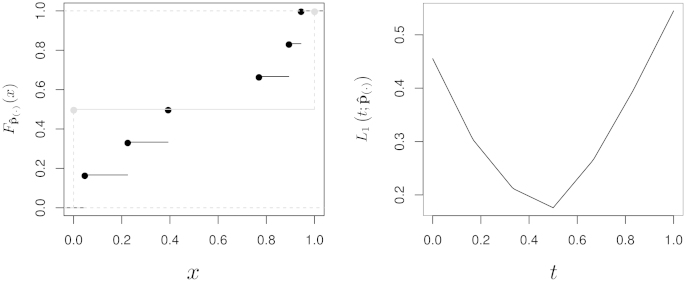
The CDFs $F_{{\hat{\text{p}}}_{\left(\cdot \right)}}$ and $F_{{\tilde{\text{p}}}_{\left(\cdot \right)}}(\hat{t})$, respectively in black and grey (left), and the $L_{1}(t;{\hat{\text{p}}}_{\left(\cdot \right)})$ norm (right) from Example 1.

The *L*_1_ norm takes the form

(16) $$L_{1}(t;{\hat{\text{p}}}_{\left(\cdot \right)})=|0-t|(0.0460-0)+\left|\frac{1}{6}-t\right|(0.2242-0.0460)+\left|\frac{2}{6}-t\right|(0.3921-0.2242)+\left|\frac{3}{6}-t\right|(0.7689-0.3921)+\left|\frac{4}{6}-t\right|(0.8935-0.7689)+\left|\frac{5}{6}-t\right|(0.9439-0.8935)+|1-t|(1-0.9439)$$

and is minimised for $\hat{t}=0.4999816$. Therefore, an edge is deemed significant if its confidence is strictly greater than $F_{{\hat{\text{p}}}_{\left(\cdot \right)}}^{-1}(0.4999816)=0.3921$, or, equivalently, if it has confidence of at least 0.7689; only (*A*, *D*), (*B*, *D*) and (*C*, *D*) satisfy this condition (Fig. 2).

## 3 Simulation results

We tested the proposed approach on synthetic data sets using three established performance measures: *sensitivity*, *specificity* and *accuracy*. *Sensitivity* is given by the proportion of edges of the true network structure that have been correctly identified as significant. *Specificity* is given by the proportion of the edges missing from the true network structure that have been correctly identified as non-significant. *Accuracy* is given by the proportion of edges correctly identified as either significant or non-significant over the set of all possible edges. To that end, we generated 400 data sets of varying sizes (100, 200, 500, 1000, 2000, 5000, 10,000 and 20,000) from three discrete BNs commonly used as benchmarks:

• the ALARM network [28], a network designed to provide an alarm message system for intensive care unit patient monitoring. Its true structure is composed by 37 nodes and 46 edges (of 666 possible edges), and its probability distribution has 509 parameters;
• the HAILFINDER network [29], a network designed to forecast severe summer hail in northeastern Colorado. Its true structure is composed by 56 nodes and 66 edges (of 1540 possible edges), and its probability distribution has 2656 parameters;
• the INSURANCE network [30], a network designed to evaluate car insurance risks. Its true structure is composed by 27 nodes and 52 edges (of 351 possible edges), and its probability distribution has 984 parameters.

Three different structure learning algorithms were considered:

• the Incremental Association Markov Blanket (IAMB) constraint-based algorithm [31]. IAMB was used to learn the Markov blanket of each node as a preliminary step to reduce the number of its candidate parents and children; a network structure satisfying these constraints is then identified as in the Grow–Shrink algorithm [32]. Conditional independence tests were performed using a shrinkage mutual information test [33] with *α* = 0.05. Such a test, unlike the more common asymptotic *χ*^2^ mutual information test, is valid and has been shown to work reliably even on small samples. An *α* = 0.01 was also considered; however, the results were not significantly different from *α* = 0.05 and will not be discussed separately in this paper;
• the Hill Climbing (HC) score-based algorithm with the Bayesian Dirichlet equivalent uniform (BDeu) score function, the posterior distribution of the network structure arising from a uniform prior distribution [7]. The equivalent sample size was set to 10. This is the same approach detailed in Friedman et al. [16], although they considered only 100 (instead of 500) bootstrap samples for each scenario;
• the Max–Min Hill Climbing (MMHC) hybrid algorithm [13], which combines the Max–Min Parents and Children (MMPC) and HC. The conditional independence test used in MMPC and the score functions used in HC are the ones illustrated in the previous points.

The performance measures were estimated for each combination of network, sample size and structure learning algorithm as follows:

1. a sample of the appropriate size was generated from either the ALARM, the HAILFINDER or the INSURANCE network;
2. we estimated the confidence values $\hat{\text{p}}$ for all possible edges from 200 and 500 nonparametric bootstrap samples. Since results are very similar, they will be discussed together;
3. we estimated the confidence threshold $\hat{t}$, and identified significant and non-significant edges in the network. Note that the direction of the edges present in the network structure is effectively ignored, because the proposed approach focuses only those edges’ presence. Significant edges were then used to build an averaged network structure;
4. we computed sensitivity, specificity and accuracy comparing the averaged network structure to the true one, which is known from the literature.

These steps were repeated 50 times in order to estimate both the performance measures and their variability.

All the simulations and the thresholds estimation were performed with the bnlearn package [34,35] for R [36], which implements several methods for structure learning, parameter estimation and inference on BNs (including the approach proposed in Section 2).

**Fig. 3:**
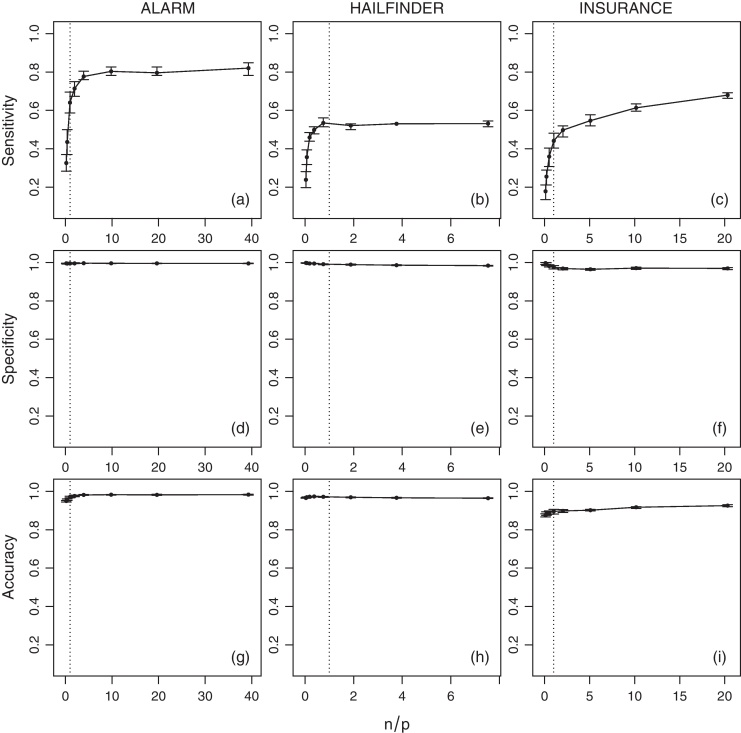
Average sensitivity, specificity and accuracy of IAMB for the ALARM, HAILFINDER and INSURANCE networks over *n*/*p*. Bars represent 95% confidence intervals, and the dotted vertical line is *n* = *p*.

The average values of sensitivity, specificity, accuracy and $\hat{t}$ for the networks across various sample sizes (*n*) are shown in Figs. 3 (IAMB), 4 (HC) and 5 (MMHC). Since the number of parameters is non-constant across the networks, a normalised ratio of the size of the generated sample to the number of parameters of the network (i.e. *n*/*p*) is used as a reference instead of the raw sample size (i.e. *n*). Intuitively, a sample of size of *n* = 1000 may be large enough to estimate reliably a small network with few parameters, say *p* = 100, but it may be too small for a larger network with *p* = 10, 000. On a related note, denser networks (i.e. networks with a large number of edges compared to the number of nodes) usually have a higher number of parameters than sparser ones (i.e. networks with few edges).

Several interesting trends emerge from the estimated quantities. As expected, sensitivity increases as the sample size grows. This provides an empirical verification that the combination of HC and BDe is indeed consistent, as proved by Chickering [23]. No analogous result exists for IAMB or MMHC, although intuitively their sensitivity should improve as well with the sample size due to the consistency of the conditional independence tests used by those algorithms. Moreover, even when *n*/*p* is extremely low a substantial proportion of the network structure can be correctly identified. When *n*/*p* is at least 0.2 (i.e. 1 observation every 5 parameters), HC successfully recovers from about 50% (for ALARM and INSURANCE) to 75% (for HAILFINDER) of the true network structure. In contrast, IAMB and MMHC successfully recover from about 45% to 50% of HAILFINDER, but only about 26% to 40% of ALARM and 19% to 30% of INSURANCE. This difference in performance can be attributed to the sparsity-inducing effect of shrinkage tests [37], which increase specificity at the cost of sensitivity. For values of *n*/*p* greater than 1 (i.e. more observations than parameters) the increase in sensitivity slows down for all combinations of networks and algorithms, reaching a plateau.

Overall, sensitivity seems to have an hyperbolic behaviour, growing very rapidly for *n*/*p* ⩽ 1 and then converging asymptotically to 1 for *n*/*p* > 1. Thus we expect it to increase linearly on a log(*n*/*p*) scale. The slower convergence rate observed for the INSURANCE network compared to the other two networks is likely to be a consequence of its high edge density (1.92 edges per node) relative to ALARM (1.24) and HAILFINDER (1.17). Slower convergence may also be an outcome of inherent limitations of structure learning algorithms in the case of dense networks [1,38].

Furthermore, both specificity and accuracy are close to 1 for all the networks and the sample sizes considered in the analysis, even at very low *n*/*p* ratios. Such high values are a result of the low number of true edges in ALARM, HAILFINDER and INSURANCE compared to the respective numbers of possible edges. This is true in particular for the ALARM and HAILFINDER networks. The lower values observed for the INSURANCE network can be attributed again to the inherent limitations of structure learning algorithms in modelling dense networks. The sparsity-inducing effect of shrinkage tests is again evident for both IAMB and MMHC; both specificity and accuracy actually decrease slightly as *n*/*p* grows and the influence of shrinkage decreases.

**Fig. 4:**
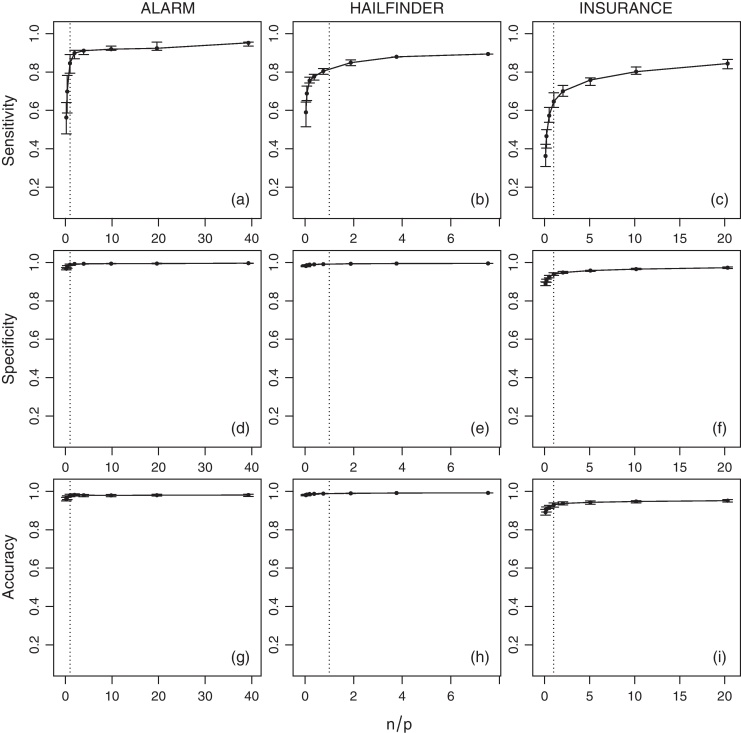
Average sensitivity, specificity and accuracy of HC for the ALARM, HAILFINDER and INSURANCE networks over *n*/*p*. Bars represent 95% confidence intervals, and the dotted vertical line is *n* = *p*.

**Fig. 5:**
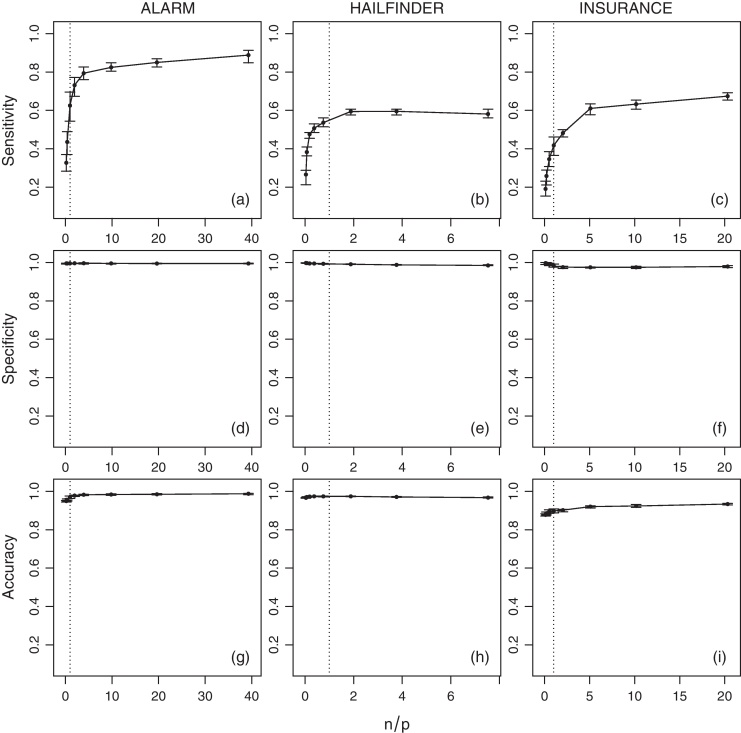
Average sensitivity, specificity and accuracy of MMHC for the ALARM, HAILFINDER and INSURANCE networks over *n*/*p*. Bars represent 95% confidence intervals, and the dotted vertical line is *n* = *p*.

**Fig. 6:**
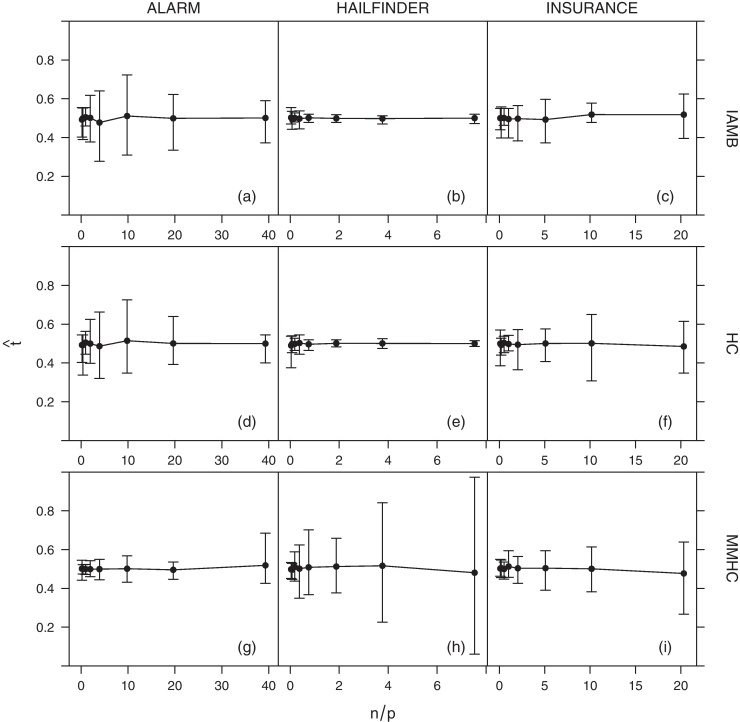
Average estimated significance threshold ($\hat{t}$) for the ALARM, HAILFINDER and INSURANCE networks over *n*/*p*. Bars represent 95% confidence intervals.

It is also important to note that, as shown in Fig. 6, the average value of the confidence threshold $\hat{t}$ does not exhibit any apparent trend as a function of *n*/*p*. In addition, its variability does not appear to decrease as *n*/*p* grows. This suggests that the optimal $\hat{t}$ depends strongly on the specific sample used in the estimation of the confidence values $\hat{\text{p}}$, even for relatively large samples. However, specificity, sensitivity and accuracy estimates appear on the other hand to be very stable (all confidence intervals shown in Figs. 3–5 are very small).

**Fig. 7:**
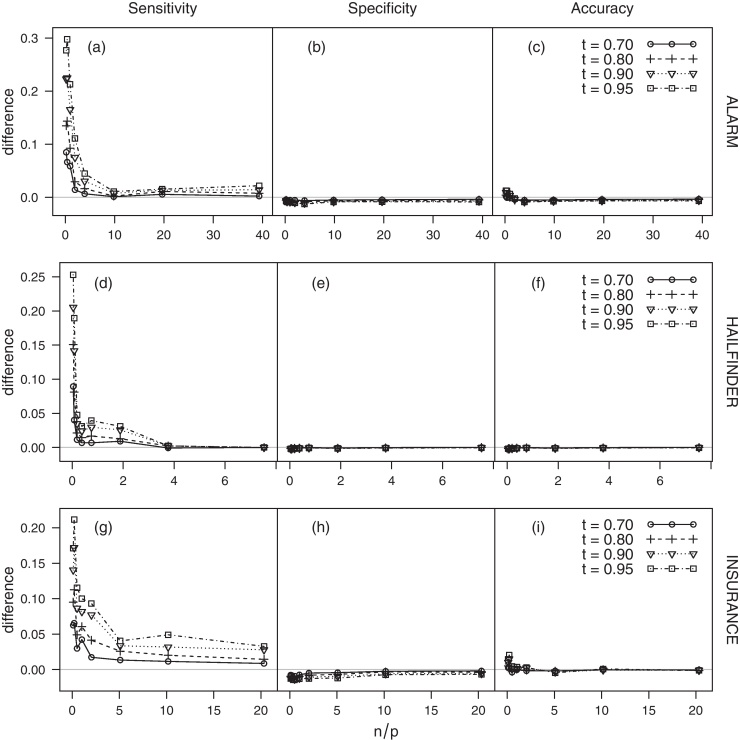
Difference in sensitivity, specificity and accuracy between the estimated threshold $\hat{t}$ and several ad hoc ones (*t* = 0.70, 0.80, 0.90, 0.95) for HC over *n*/*p*.

From Fig. 6, it is also apparent that the threshold estimate $\hat{t}$ can be significantly lower than 1 even for high values of *n*/*p*. This behaviour is observed consistently across the three networks (ALARM, HAILFINDER, INSURANCE). These results are in sharp contrast with ad hoc thresholds commonly found in the literature, which are usually large [e.g. 0.8 in 16]. A large threshold can certainly be useful in excluding noisy edges, which may result from artefacts at the measurement and dynamical levels and from finite sample-size effects. However, while a large ad hoc threshold can certainly minimise false positives, it is also expected to accentuate false negatives. Such a conservative choice can have a profound impact on the network topology, resulting in artificially sparse networks. The threshold estimator introduced in Section 2 achieves a good trade-off between incorrectly identifying noisy edges as significant and disregarding significant ones. As an example, the difference in sensitivity, specificity and accuracy between the estimated threshold $\hat{t}$ and several large, ad hoc ones (*t* = 0.70, 0.80, 0.90, 0.95) for HC is shown in Fig. 7 (the corresponding plots for IAMB and MMHC are similar, and are omitted for brevity). The threshold $\hat{t}$ systematically outperforms the ad hoc thresholds in terms of sensitivity, in particular for low values of *n*/*p*. The difference progressively vanishes as *n*/*p* grows. All thresholds have comparable levels of specificity and accuracy.

On a related note, false negatives across ad hoc thresholds may also be attributed to the fact that edges are considered as separate, independent entities as far as the choice of the threshold is concerned – i.e. a 0.99 threshold is expected to identify as significant about 1 in 100 edges in the network. However, in a biological setting the structure of the network is an abstraction for the underlying functional mechanisms; as an example, consider the signalling pathways in a transcriptional network. In such a context, edges are clearly not independent, but appear in concert along signalling pathways. This interdependence is accounted for in the proposed approach (that is based on the full set $\hat{\text{p}}$ of estimated confidence values), but it is not commonly considered in choosing ad hoc thresholds. For instance, edges appearing with individual confidence values far below the [0.80, 1] range may not necessarily be identified as significant by an ad hoc threshold. However, the proposed approach recognises their interplay and correctly identifies them as significant. This aspect, along with the strong dependence between the optimal $\hat{t}$ and the actual sample the network is learned from, may discourage the use of an a priori or ad hoc confidence threshold in favour of more statistically motivated alternatives.

## 4 Applications to molecular expression profiles

In order to demonstrate the effectiveness of the proposed approach on experimental data sets, we will examine two gene expression data sets from Nagarajan et al. [20] and Sachs et al. [21]. All the analyses will be performed again with the bnlearn package. Following Imoto et al. [39], we will consider the edges of the BNs disregarding their direction when determining their significance. Edges identified as significant will then be oriented according to the direction observed with the highest frequency in the bootstrapped networks $G_{b}$. While simplistic, this combined approach allows the proposed estimator to handle the edges whose direction cannot be determined by the structure learning algorithm possibly due to score equivalent structures [40].

### 4.1 Differentiation potential of aged myogenic progenitors

In a recent study [20] the interplay between crucial myogenic (Myogenin, Myf-5, Myo-D1), adipogenic (C/EBP*α*, DDIT3, FoxC2, PPAR*γ*), and Wnt-related genes (Lrp5, Wnt5a) orchestrating aged myogenic progenitor differentiation was investigated by Nagarajan et al. using clonal gene expression profiles in conjunction with BN structure learning techniques. The objective was to investigate possible functional relationships between these diverse differentiation programs reflected by the edges in the resulting networks. The clonal expression profiles were generated from RNA isolated across 34 clones of myogenic progenitors obtained across 24-month-old mice and real-time RT-PCR was used to quantify the gene expression. Such an approach implicitly accommodates inherent uncertainty in gene expression profiles and justified the choice of probabilistic models.

In the same study, the authors proposed a non-parametric resampling approach to identify significant functional relationships. Starting from Friedman's definition of confidence levels (Eq. (1)), they computed the *noise floor distribution* $\hat{\text{f}}=\{{\hat{f}}_{1},{\hat{f}}_{2},\ldots ,{\hat{f}}_{k}\}$ of the edges by randomly permuting the expression of each gene and performing BN structure learning on the resulting data sets. An edge *e*_*i*_ was deemed significant if ${\hat{p}}_{i}>\max_{\left\{{\hat{f}}_{l}\in \hat{\text{f}}\right\}}{\hat{f}}_{l}$. In addition to revealing several functional relationships documented in literature, the study also revealed new relationships that were immune to the choice of the structure learning techniques. These results were established across clonal expression data normalised using three different housekeeping genes and networks learned with three different structure learning algorithms.

The approach presented in [20] has two important limitations. First, the computational cost of generating the noise floor distribution may discourage its application to large data sets. In fact, the generation of the required permutations of the data and the subsequent structure learning (in addition to the bootstrap resampling and the subsequent learning required for the estimation of $\hat{\text{p}}$) essentially doubles the computational complexity of Friedman's approach. Second, a large sample size may result in an extremely low value of $\max(\hat{\text{f}})$, and therefore in a large number of false positives.

**Fig. 8:**
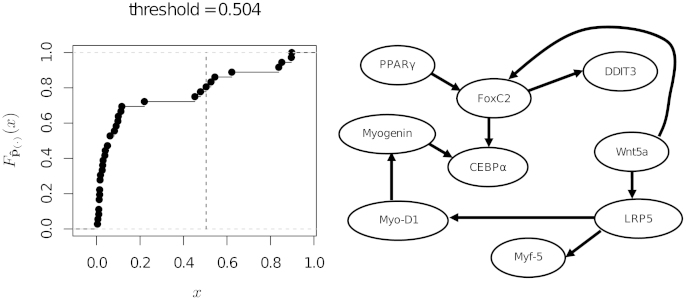
The empirical CDF $F_{{\hat{\text{p}}}_{\left(\cdot \right)}}$ for the myogenic progenitors data from Nagarajan et al. [20] (on the left), and the network structure resulting from the selection of the significant edges (on the right). The vertical dashed line in the plot of $F_{{\hat{\text{p}}}_{\left(\cdot \right)}}$ represents the threshold $F_{{\tilde{\text{p}}}_{\left(\cdot \right)}}^{-1}(\hat{t})$.

In the present study, we re-investigate the myogenic progenitor clonal expression data normalised using housekeeping gene GAPDH with the approach outlined in Section 2 and the IAMB algorithm. It is important to note that this strategy was also used in the original study [20], hence its choice. The order statistic ${\hat{\text{p}}}_{\left(\cdot \right)}$ was computed from 500 bootstrap samples. The empirical CDF $F_{{\hat{\text{p}}}_{\left(\cdot \right)}}$, the estimated threshold and the network with the significant edges are shown in Fig. 8.

All edges identified as significant in the earlier study [20] across the various structure learning techniques and normalisation techniques were also identified by the proposed approach (see Fig. 3D in [20]). In contrast to Fig. 8, the original study using IAMB and normalisation with respect to GAPDH alone detected a considerable number of additional edges (see Fig. 3A in [20]). Thus it is quite possible that the approach proposed in this paper reduces the number of false positives and spurious functional relationships between the genes. Furthermore, the application of the proposed approach in conjunction with the algorithm from Imoto et al. [39] reveals directionality of the edges, in contrast to the undirected network reported by Nagarajan et al. [20].

### 4.2 Protein signalling in flow cytometry data

In a landmark study, Sachs et al. [21] used BNs for identifying causal influences in cellular signalling networks from simultaneous measurement of multiple phosphorylated proteins and phospholipids across single cells. The authors used a battery of perturbations in addition to the unperturbed data to arrive at the final network representation. A greedy search score-based algorithm that maximises the posterior probability of the network [7] and accommodates for variations in the joint probability distribution across the unperturbed and perturbed data sets was used to identify the edges [41]. More importantly, significant edges were selected using an arbitrary significance threshold of 0.85 (see Fig. 3, [21]). A detailed comparison between the learned network and functional relationships documented in the literature was presented in the same study.

**Fig. 9:**
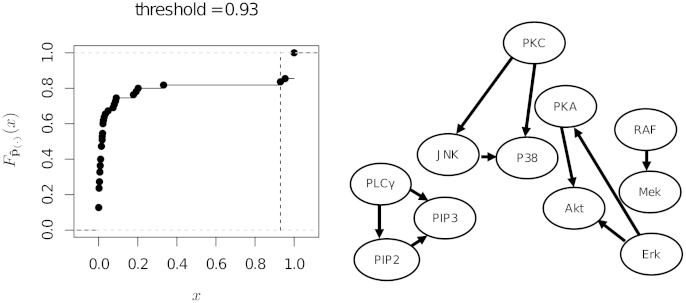
The empirical CDF of ${\hat{\text{p}}}_{\left(\cdot \right)}$ for the flow cytometry data from Sachs et al. [21] (on the left), and the network structure resulting from the selection of the significant edges (on the right). The vertical dashed line in the plot of $F_{{\hat{\text{p}}}_{\left(\cdot \right)}}$ represents the threshold $F_{{\tilde{\text{p}}}_{\left(\cdot \right)}}^{-1}(\hat{t})$.

We investigated the performance of the proposed approach in identifying significant functional relationships from the same experimental data. However, we limit ourselves to the data recorded without applying any molecular intervention, which amount to 854 observations for 11 variables. We compare and contrast our results to those obtained using an arbitrary threshold of 0.85. The combination of perturbed and non-perturbed observations studied in Sachs et al. [21] cannot be analysed with our approach, because each subset of the data follows a different probability distribution and therefore there is no single “true” network $G_{0}$. Analysis of the unperturbed data using the approach presented in Section 2 reveals the edges reported in the original study. The resulting network is shown in Fig. 9 along with $F_{{\hat{\text{p}}}_{\left(\cdot \right)}}$ and the estimated threshold. From the plot of $F_{{\hat{\text{p}}}_{\left(\cdot \right)}}$ we can clearly see that significant and non-significant edges present widely different levels of confidence, to the point that any threshold between 0.4 and 0.9 results in the same network structure. This, along with the value of the estimated threshold (${\hat{p}}_{\left(i\right)}⩾0.93$), shows that the noisiness of the data relative to the sample size is low. In other words, the sample is big enough for the structure learning algorithm to reliably select the significant edges. The edges identified by the proposed method were the same as those identified by [21] using general stimulatory cues excluding the data with interventions (see Fig. 4A in [21], Supplementary information). In contrast to [21], using Imoto et al. [39] approach in conjunction with the proposed thresholding method we were able to identify the directions of the edges in the network. The directions correlated with the functional relationships documented in literature (Table 3, [21], Supplementary information) as well as with the directions of the edges in the network learned from both perturbed and unperturbed data (Fig. 3, [21]).

## 5 Conclusions

Graphical models and network abstractions have enjoyed considerable attention across the biological and medical communities. Such abstractions are especially useful in deciphering the interactions between the entities of interest from high-throughput observational data. Classical techniques for identifying significant edges in the resulting graph rely on ad hoc thresholding of the edge confidence estimated from across multiple independent realisations of networks learned from the given data. Large ad hoc threshold values are particularly common, and are chosen in an effort to minimise noisy edges in the resulting network. While useful in minimising false positives, such a choice can accentuate false negatives with pronounced effect on the network topology. The present study overcomes this caveat by proposing a more straightforward and statistically motivated approach for identifying significant edges in a graphical model. The proposed estimator minimises the *L*_1_ norm between the CDF of the observed confidence levels and the CDF of their asymptotic, ideal configuration. The effectiveness of the proposed approach is demonstrated on three synthetic data sets [28–30] and on gene expression data sets across two different studies [20,21]. However, the approach is defined in a more general setting and can be applied to many classes of graphical models learned from any kind of data.

## Footnotes

1 The probabilities $\hat{P}(e_{i})$ are in fact an estimator of the expected value of the {0, 1} random vector describing the presence of each possible edge in $G_{0}$. As such, they do not sum to one and are dependent on one another in a nontrivial way.
